# Connecting the Dots: From an Easy Method to Computerized Species Determination

**DOI:** 10.3390/insects8020052

**Published:** 2017-05-18

**Authors:** Senta Niederegger, Klaus-Peter Döge, Marcus Peter, Tobias Eickhölter, Gita Mall

**Affiliations:** 1 Institute of Legal Medicine, University Hospital Jena, Am Klinikum 1, 07747 Jena, Germany; Gita.Mall@med.uni-jena.de; 2 Department of Electrical Engineering and Information Technology, University of Applied Sciences Jena, Carl-Zeiss-Promenade 2, 07745 Jena, Germany; Klaus-Peter.Doege@eah-jena.de (K.-P.D.); Marcus.Peter@stud.eah-jena.de (M.P.); eickhoelter.tobias@googlemail.com (T.E.)

**Keywords:** forensic entomology, muscle attachment sites, species determination, image processing, correlation, knowledge base

## Abstract

Differences in growth rate of forensically important dipteran larvae make species determination an essential requisite for an accurate estimation of time since colonization of the body. Interspecific morphological similarities, however, complicate species determination. Muscle attachment site (MAS) patterns on the inside of the cuticula of fly larvae are species specific and grow proportionally with the animal. The patterns can therefore be used for species identification, as well as age estimation in forensically important dipteran larvae. Additionally, in species where determination has proven to be difficult—even when employing genetic methods—this easy and cheap method can be successfully applied. The method was validated for a number of Calliphoridae, as well as Sarcophagidae; for Piophilidae species, however, the method proved to be inapt. The aim of this article is to assess the utility of the MAS method for applications in forensic entomology. Furthermore, the authors are currently engineering automation for pattern acquisition in order to expand the scope of the method. Automation is also required for the fast and reasonable application of MAS for species determination. Using filters on digital microscope pictures and cross-correlating them within their frequency range allows for a calculation of the correlation coefficients. Such pattern recognition permits an automatic comparison of one larva with a database of MAS reference patterns in order to find the correct, or at least the most likely, species. This facilitates species determination in immature stages of forensically important flies and economizes time investment, as rearing to adult flies will no longer be required.

## 1. Introduction

Blowflies often start the biological clock for postmortem interval (PMI) calculation in forensic entomology. They might approach a body within the first hours after death [1,2,3] for oviposition. The age of the oldest blowfly found on a body or carcass is therefore a good estimate for the minimum postmortem interval or minimum exposure time [4,5,6]. Laboratory experiments growing larvae of known species under controlled temperature regimes showed that age correlates to the body length of the animals [7,8,9,10,11]. However, even though the larvae of different species look extremely similar, they might grow to different body lengths in the same time and under identical conditions [11]. Species and age determination can therefore be a great challenge when a conglomerate of unidentified larvae is collected from a body in the field.

A common method for species determination is to allow collected larvae to pupate and hatch, and to determine the species by morphological features of the imago [3,12]. This is very time-consuming and requires living larvae. Other methods based on light microscopy rely on the morphological characteristics of the larvae, like the shape of the cephalopharyngeal skeleton (CPS), spiracles, or spinebands [13,14,15,16,17,18,19,20,21,22]. These methods can be hindered by the fragmentation or discoloration of the specimen by poor preservation. Technically advanced approaches aim toward DNA-based species identification using molecular biological methods [23,24,25] or electron microscopy [26,27]. Requiring cost-intensive and specialized equipment, these methods might not be applicable in every laboratory.

## 2. Approach from the Biological Perspective

**Figure 1:**
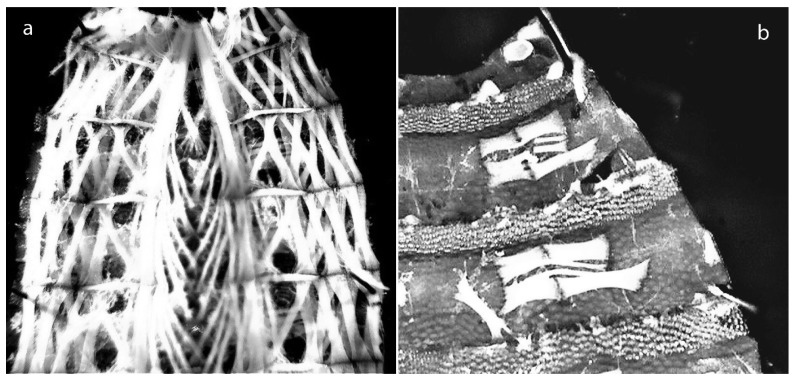
(**a**) Opened larva with complete musculature; (**b**) The longitudinal muscles were removed to reveal the underlying transversal muscle groups.

A fast and easy method, suited for all larval instars and even applicable to a fragmented or discolored specimen, was developed in 2012: While attempting to locate trichoid sensilla, round structures were detected on the inside of the cuticula of a number of brachyceran larvae. Further investigation revealed that they were attachment sites for transversal body wall muscles. The thoracic segments, as well as the first and last abdominal segments, each possess a unique muscle arrangement, while the remaining abdominal segments share an identical pattern [28,29]. The body wall musculature of many larvae is composed of longitudinal, diagonal, and transversal muscles (Figure 1a). Larval flies lack circumferential muscles as antagonists to the longitudinal muscles [30]. The vertical displacement of the segments is therefore probably supported by the transversal muscles which are located in the external layer, where they form “blocks” in each segment and are directly attached to the cuticula [31] (Figure 1b).

### 2.1. Species and Age Determination

**Figure 2:**
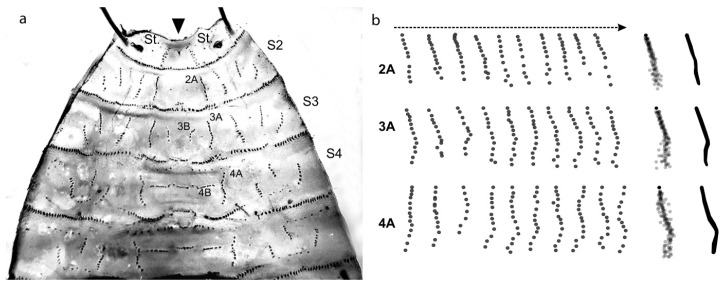
(**a**) All muscles were removed from the transparent cuticle, and attachment sites were colored with Coomassie brilliant blue. Patterns are laterally symmetrical. Investigated muscle groups labelled 2A, 3A, 3B, and 4A, 4B, according to segments; (**b**) Patterns of ten larvae of the same species photographed, with attachment sites marked with semitransparent dots. Resulting rows were stacked and areas with a high degree of overlap are marked in black.

The muscle attachment sites (MAS) of these transversal muscles could easily be visualized by removing the muscles from the cuticle and subsequent staining with Commassie brilliant blue. The analysis of MAS revealed that the structures are arranged in rows and form distinct patterns (Figure 2a). Ten larvae of each species were prepared in this fashion and the pictures were recorded. A small number of row subsets located at the center of the last two thoracic and the first abdominal segment (Segments 2–4) were manually charted. Correlating rows were stacked and condensed into row patterns (Figure 2b) and described for a few forensically important species of blowflies (Diptera: Calliphoridae) *Calliphora vomitoria*, *C. vicina*, *Lucilia sericata* [32], *Protophormia terraenovae* [33], and *Phormia regina* [34]. The results confirmed interspecific variation which allowed for the species differentiation of these five species. There can be variation in the number of attachment points composing a row in larvae of the same age and species. Such variability is common in biological systems and also present in other morphological characteristics, such as the ratio of the length of the mouth hook base and the distance between the base and the tip [35] or the length and width of the posterior spiracle discs [36]. The reason for the divergence in MAS numbers remains unclear, but does not affect the usability of the method.

**Figure 3:**
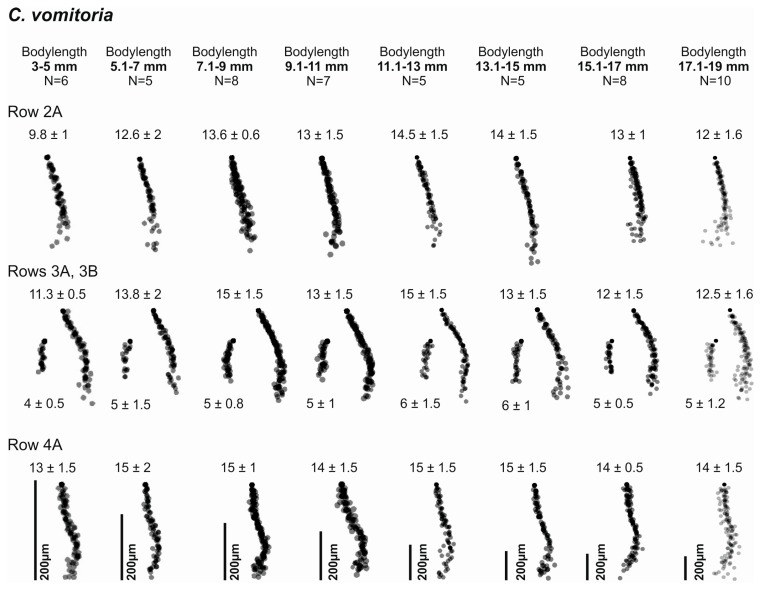
Pattern consistency during growth of *C. vomitoria.*

The observation of larvae in different developmental stages further showed that the basic central muscle attachment site patterns are conserved throughout development. The length of each row, however, increases with the body length of the maggot (Figure 3). The method could thus not only be used for species determination, but also for age determination [33].

### 2.2. Genus Lucilia

**Figure 4:**
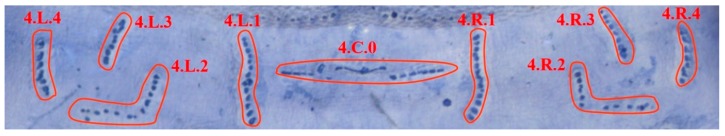
New labeling in segment 4 shown for *Calliphora vicina.*

To further challenge the method, six closely related and forensically relevant species of the genus Lucilia were tested [37]. In order to achieve reliable results, the set of analyzed MAS patterns had to be enlarged. While isolated central patterns yielded adequate results in the first experiments [32], it became necessary to include all rows present on segments two, three, and four. The labeling of MAS rows was changed accordingly (Figure 4, shown for *Calliphora vicina*).

**Figure 5:**
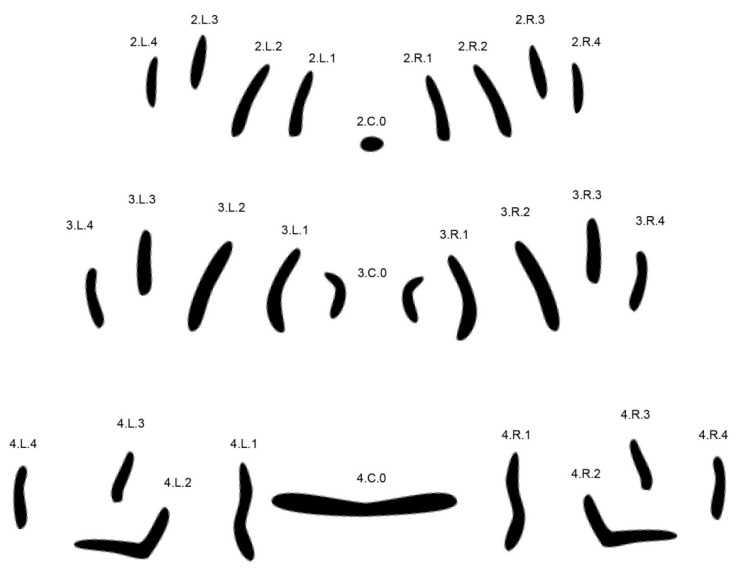
Genus pattern for Lucilia composed of 76 individual MAS patterns.

A joint genus pattern (Figure 5) could be found by combining pictures of all six Lucilia species tested (*L. ampullacea, L. caesar, L. illustris, L. richardsi, L. sericata*, and *L. silvarum*). Each species differed from the genus pattern in a characteristic way. This allowed for the composition of distinct species patterns for each of the six species. For *L. illustris* and *L. caesar*—where the species determination of larvae was reported to be difficult [38,39,40] or impossible [41], even with genetic methods [42]—MAS patterns were distinctive [37].

### 2.3. Genus Sarcophaga

**Figure 6:**
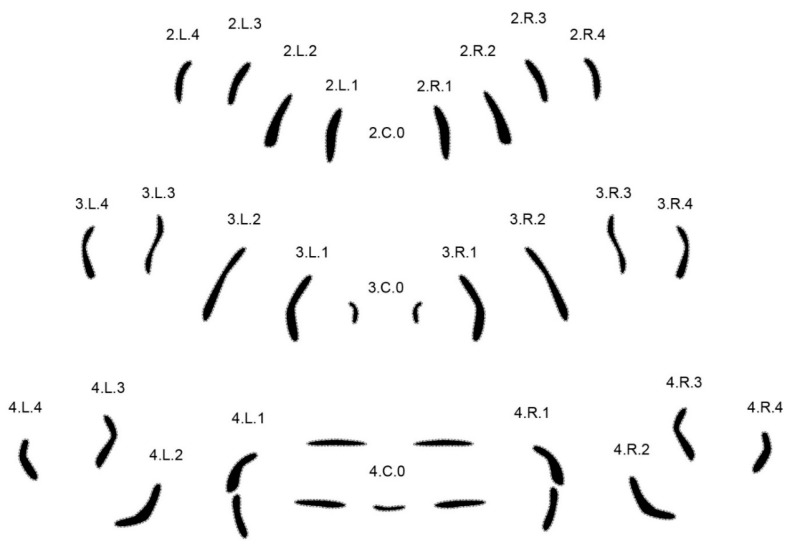
Genus pattern for Sarcophaga composed of 50 individual MAS patterns.

Flesh fly (*Sarcophaginae*) larvae, including the genus Sarcophaga, can easily be recognized from the large and deep spiracular cavity on the last segment [43]. Species identification, however, is much more difficult. Therefore, the next step in the development of the method was designed for five species of the genus Sarcophaga from five different subgenera. *Sarcophaga albiceps*, *S. argyrostoma*, *S. caerulescens*, *S. melanura*, and *S. similis* were previously found to be frequent visitors and successful colonizers of large animal carrion and human corpses [44,45,46,47,48,49,50]. As for the six Lucilia species, a genus pattern could be generated from these five Sarcophaga species (Figure 6) and species-specific pattern variations were found [51]. The previously used set of MAS did not have to be extended. The genus pattern for Sarcophaga species is distinctively different from the Lucilia genus pattern. This indicates the presence of variations between genera and families which promise to be very useful for the MAS method.

### 2.4. Family Piophilini

**Figure 7:**
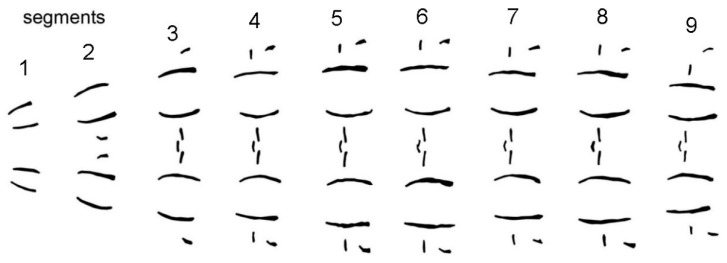
Preliminary family pattern for Piophilini.

Preliminary analyses of Piophilini species showed that their muscle equipment is clearly different from the Calliphoridae and Sarcophagidae (Figure 7). The very similar species patterns observed between the two closely related Piophilina genera and a more distantly related genus of Thyreophorina, however, suggest that the larval MAS patterns may be highly conserved among the group [52]. Sufficiently conspicuous variations for reliable species differentiation were not visible to the naked eye.

### 2.5. Next Steps

The main aim of the method is fast and reliable species identification in single, unknown larvae. A vast pool of data, as well as reference genus and species patterns, is an essential requirement for comparison. It is therefore of vital importance to optimize the process of pattern acquisition. The manual charting of patterns is very time consuming. The manual generation of reference patterns for genera and species can never be objective. An automation of the process is therefore required for a successful extension of the knowledge base of the method. It could furthermore permit the distinction of patterns with only marginal differences. A large data base, on the other hand, is reliant on efficient comparison methods to achieve useful results. The comparison of patterns for species determination necessitates a degree of expertise and using an extensive database would be very time-consuming. Customized software is therefore the only option to allow for a widespread and successful application of the MAS method.

## 3. Problem Analysis from the Image Processing Perspective

In the following, image processing will be examined as a potential method of refinement. Digital image processing has continuously developed over the last decades. However, for this method, it was necessary to test the general applicability before process development. This is mainly due to the variability of image information and was done in a series of example images of different species. Images of larvae belonging to instar L3 were exclusively used for all of the following tests.

**Figure 8:**
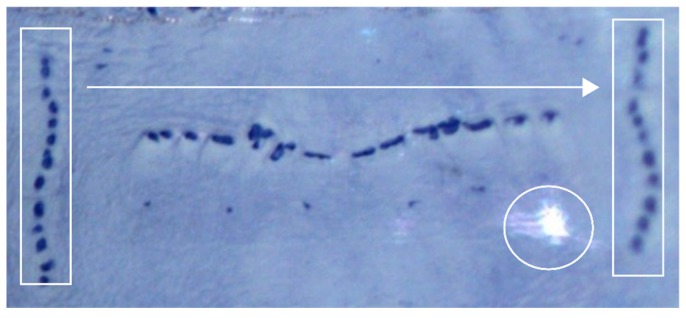
Examples of image information variability in the fourth segment of *Lucilia ampullacea*. Variability of individual patterns (rectangle), reflections (circle), and variation of resolutions (arrow) are shown.

The evaluation showed that patterns within the same segments of a species are recognizable, but variable. Neither the number of spots nor the general shape is predictable, which is illustrated by the rectangle in Figure 8. For a human observer, the differentiation of species is still possible [32,37]. For an automatized procedure, variability presents a challenge due to missing contextualization. In addition to the variability of patterns, differences in the sizes of larvae need to be taken into account in automated processes.

Furthermore, transforming three-dimensional objects into two-dimensional images leads to differences in imagesharpness, which is indicated by the arrow in Figure 8. The image also shows significant differences in brightness, highlighted by the circle in Figure 8, which are not immanent to the object itself, but due to bad illumination. Again, the human eye recognizes these effects as interferences and is still able to deduce the underlying information. In an automated process, however, these effects of interferences need to be explicitly addressed. Consequently, for reliable image capturing, it must be adjusted to fit the analytical process. This also includes the parallel alignment of the pictorial object to the vertical image edge.

Noticing the need of expert knowledge for the identification of relevant patterns, as well as for the elimination of interferences, the utilization of a knowledge base was inevitable for species determination in this project.

Table 1 summarizes the postulated assessment of starting conditions.

**Figure 9:**
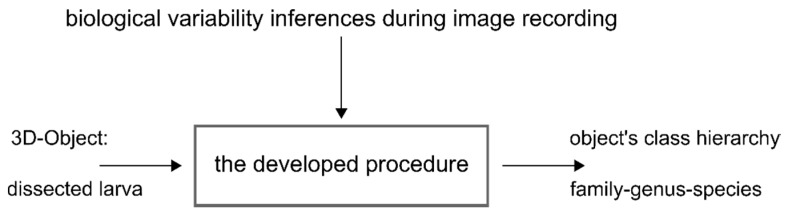
Specification of problem definition and primary influential factors.

It appears worthwhile to enhance the task of species determination using methods of image processing. From the applications point of view, the task is to assign a three-dimensional object, the larva, to its class hierarchy, consisting of family, genus, and species. Biological variability and interferences of image processing need to be taken into consideration (Figure 9).

## 4. The Image Processing Software

**Figure 10:**
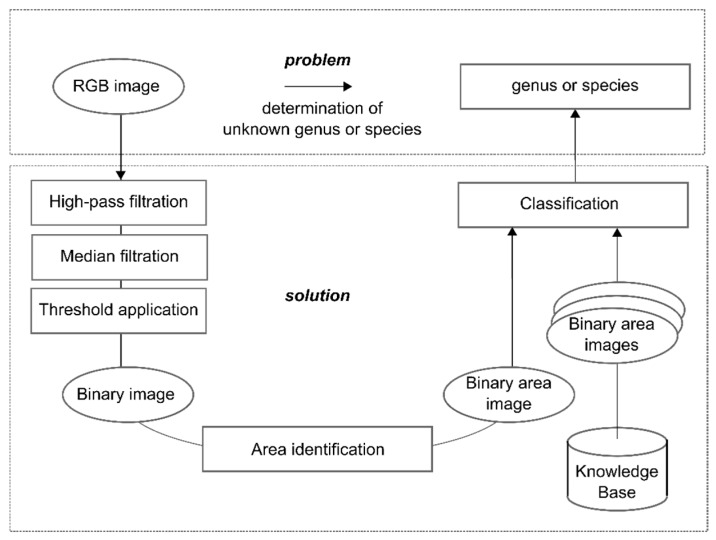
Solution from an image processing perspective.

Building on the generally applicable hierarchy of image processing operations [53], the steps shown in Figure 10 are necessary to solve the task using methods of image processing.

### 4.1. Filtration

**Figure 11:**
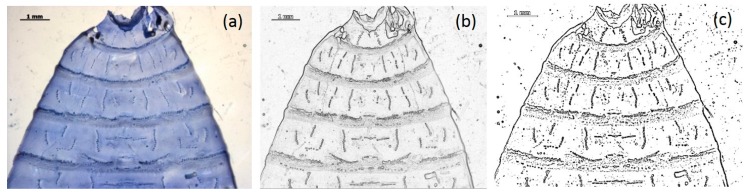
Steps of the filtration process: Original image (**a**); high-pass filtration (**b**); and binary image (**c**).

The aim of filtration processes (Figure 11) is to emphasize the patterns which are to be used for classification and to remove any image perturbations. In other words, interfering signals should be suppressed. Starting with the original image (a), three steps are necessary to achieve this goal: high-pass filtration (b), median filtration as preparation for the generation of a binary image (c).

#### 4.1.1. High-Pass Filtration to Emphasize the Pattern

The prerequisite for automated identification is a good perceptibility of the patterns against the background. Good visibility is necessary to obtain the characteristics of the desired signal. The light/dark boundary of the image allows for the separation of frequencies. In image processing, these boundaries are called “edges”.

The image undergoing the filtration process is considered to be a two-dimensional signal. The edges correspond to high frequencies, which need to be emphasized. In signal processing, this can be achieved using high-pass filtration. A Sobel filter was chosen for the present application. This differentiation filter of the 1st order implements an approximation of the first derivative, and smoothing of the image in the orthogonal direction to the derivative.

The filter operation consists of 3 × 3 matrices for the *x* and *y* direction.

$$S_{x}=\left[\begin{array}{ccc} -1 & 0 & 1\\ -2 & 0 & 2\\ -1 & 0 & 1 \end{array}\right],\;S_{y}=\left[\begin{array}{ccc} -1 & -2 & -1\\ 0 & 0 & 0\\ 1 & 2 & 1 \end{array}\right]$$

The filtration involves convolution of the image matrix A with the operators ***S_x_*** and ***S_y_,*** and results in the filtered image composed of the sum of the individual convolution results.

$$A_{xy}=\sqrt{{\left(S_{x}*A\right)}^{2}+{\left(S_{y}*A\right)}^{2}}$$

#### 4.1.2. Median Filtration to Reduce Image Noise

The high-pass filtration emphasized the desired signal. Since the noise in the image also consists of high frequency sections, they, too, are now emphasized. They must therefore be suppressed separately using a median filter. The median for each pixel with its adjacent neighbor is calculated, resulting in nine values. The median value in this list represents the new brightness of the filtered pixel. At the image margin, the number of neighbors is reduced to five, and at the corner, to three.

#### 4.1.3. Generating a Binary Image to Compare with the Knowledge Base

The knowledge base consists of binary images. The patterns that are to be compared must therefore be converted into the same format. After the application of steps 1 and 2, the image still includes 256 brightness levels. The application of a static or dynamic threshold allows for the conversion of these brightness levels into binary values. A static threshold with a dynamic component was used in this case. The dynamic component is derived from the average brightness of all pixels. The static component is given by an offset value, which was set to an initial value of 20. This is a proper value for well exposed and sharp images and was determined empirically.

### 4.2. Area Identification

The subdivision of individual patterns from the filtered image is currently done semi-automatically. The user identifies the relevant areas by manually drawing the outline as free form. Each selection containing a single pattern is then automatically stored as an individual image and labelled accordingly. The resulting images are further filtered for disruptive pixel groups, removing small groups of pixels that could negatively influence classification. The last step of the process is the automatic cropping and scaling of the pattern images to 100 pixels in height. This adjustment takes account of size differences in larvae according to species and the state of development.

Area identification generates up to nine images per segment. For each animal, the images of all segments analyzed are stored in one extensible-markup-language-file (XML-file), sorted according to the location of the pattern in each segment. These files provide the base for the following classification tasks and enable visual verification of the image processing steps.

### 4.3. Classification via Knowledge Base

**Figure 12:**
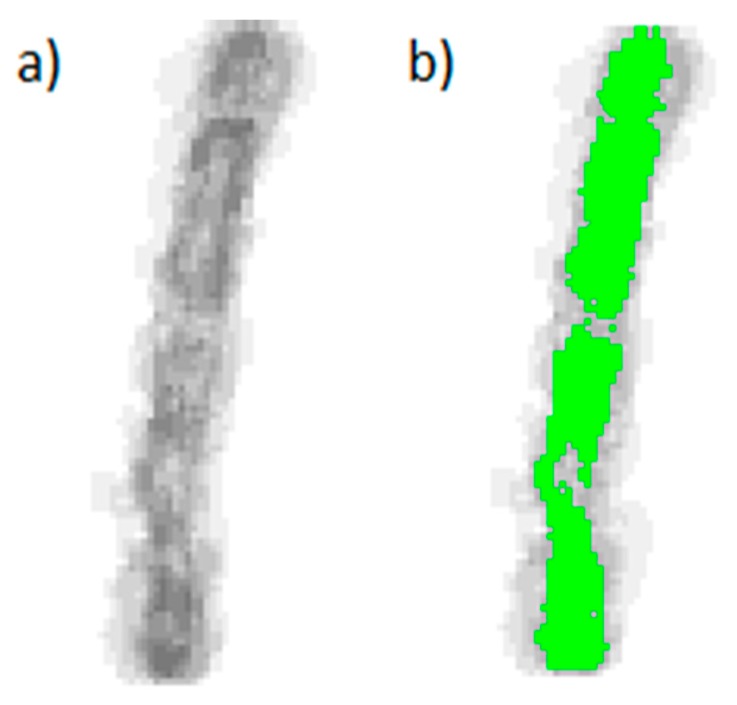
Layering of several individual patterns (**a**) to generate a reference pattern (**b**).

The classification of the patterns requires the generation and application of a knowledge base. Sample images of larvae belonging to 14 distinct species (Table 2) were selected and run through the steps described in Section 4.1 (Filtration). To achieve a high level of resolution, the smallest possible meaningful elements, i.e., the individual patterns (as shown in Figure 12 and previously explained in Figure 8), were chosen to construct the knowledge base.

Reference patterns for each species are generated by user controlled iterative layering of the resulting sample images (Figure 12).

The user is able to remove distorting images or to manually correct images. Such interventions are needed, especially when the patterns are heavily twisted or have an unusual size. The construction of the reference patterns is therefore strongly influenced by the experience and knowledge of the person conducting the process. Consequently, the knowledge base represents expert know-how.

In analogy to the analysis images, reference images need to be cropped and scaled to 100 pixels in height and saved as XML-files. These files constitute the base for the computational implementation of the knowledge base.

To ultimately be able to perform species determination in images from unknown larvae, the classification method needs to be detailed. The calculation of a normative cross-correlation coefficient [54] between the patterns of the binary image *A_xy_* and the reference object *H_xy_* is the basis of the classification used here.

$$k\left(x,y\right)=\frac{\sum_{j,i}H_{xy}\left(i,j\right)A_{xy}\left(x+i,y+j\right)}{\sqrt{H_{xy}{\left(i,j\right)}^{2}}\sqrt{A_{xy}{\left(x+i,y+j\right)}^{2}}}$$

**Figure 13:**
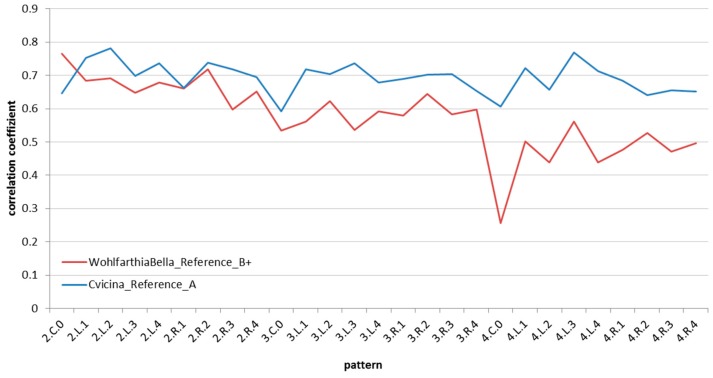
Graphs of correlation for two patterns.

*k*_max_ is determined for each pattern, resulting in a characteristic gradient for each reference image (Figure 13).

An average is calculated based on this gradient and used for evaluation. The reference image with the highest average correlation to the analysis image represents the fly species that the larva in question supposedly belongs to.

## 5. Results

The original images of larvae from 14 known species were sorted into three groups for the test run of the software. Group A consisted of high quality images containing a full set of patterns. Group B contained acceptable images missing single patterns. Group C contained images of poor quality in which a large share of the patterns could not be extracted for reasons of poor lighting, insufficient contrast, or discolorations.

The images from group A and B were used to compile references for each species, where further differentiations were made: Class A references were exclusively composed of group A images. If the number of A images was insufficient for the generation of reliable reference patterns, class B references were composed either of group B images or a mix of A and B images. If necessary, C references were composed of B and C images.

While generating the knowledge base, single images had to be removed from the layering due to significant contortion. Two species (*Sacrophaga melanura* and *Lucilia sericata)* had to be excluded altogether due to the lack of suitable images. Patterns were visible, but could only be extracted in poor quality or not at all. For some species, groups A and B could be employed, and for others, only group B could be used. The test compared each image to each reference.

### 5.1. Results of Species Determination

The three references with the highest average correlation coefficients, as well as the three references with the highest sum of correlation coefficients, were selected as the results. A total of 134 larvae from 14 different species were analyzed, of which 124 (92.5%) could be identified correctly (Table 2).

### 5.2. Results of Genus Determination

The three references with the highest correlation coefficient are further investigated. The result is conceded as true if the three references belong to the same genus; otherwise, no genus could be determined. The test included 97 larvae from 13 different species, where 68 larvae (70%) were identified correctly (Table 3).

One requirement is mandatory for genus determination: The references of the same genus must be more similar to one another than to those of any other genus. To validate this requirement, all references were compared to one another. For each reference, the three references with the highest correlation coefficients are given (extract in Table 4). Each genus was represented by at least four species. For a correct determination, all three correlating reference species must therefore be members of the same genus. 

The highlighted fields indicate samples in which this could not be achieved. This suggests that differing genus references might display more similarities to the investigated sample than references of the corresponding genus. The identification of the genera Lucilia and Sacrophaga proved to be difficult. The genus Wohlfarthia, on the other hand, could always be determined correctly (Table 4).

### 5.3. Problem Analysis

All of the following problems (Table 5) were identified during the process. The problems do not generally occur and combinations are possible.

## 6. Discussion and Outlook

The aim of our work was to improve the method of MAS pattern recognition for species and age identification in larval instars of Diptera. The MAS method is based on the comparison of an unknown pattern (of a larva in question) with existing reference patterns (generated from patterns of a number of known larvae). A broad data base of reference patterns is needed for this task. Facilitation of MAS pattern recognition allows for the much faster generation of reference patterns and thus, the expansion of the knowledge base.

At first glance, the use of other methods than a knowledge base sounds promising. Complex identification problems can often be solved with methods of so called artificial intelligence. This approach, however, had to be declined for the following reasons.

First, it is hardly possible to create a suitable amount of learning data to meet the claim of, e.g., a neural network. This is due to the intricate process of the manual preparation of larvae. We are talking about at least 100 perfect samples to support the learning process. In the probabilistic case of failure or a reduced accuracy of the learning process, this number would have to be increased. The second reason is the basic necessity of a coefficient of determination. The proposed method provides the average correlation coefficient, while neural networks cannot evaluate the accuracy of the results of image processing by themselves.

Entomological scientists from around the world might be needed to contribute and analyze a vast pool of known larvae for the MAS method. To facilitate this, protocols need to be established for standardization in the preparation of larvae, the production of original pictures, and the use of the software.

All hitherto existing preparations were photographed with a digital camera mounted onto the dissecting microscope. Special care was given to the vertical adjustment of the ventral midline for a better comparison of patterns. Lighting aspects, however, were of no particular importance while analyzing the pictures by hand and in small numbers. This resulted in scattered illumination on the original pictures. To avoid the editing of original pictures prior to automated analysis, a new protocol has to be developed. Pictures need to be recorded in optimal lighting conditions with combined reflected and transmitted illumination. This is possible when the cuticula is pinned to a clear base, like two-component Sylgard^®^ silicone, using very fine tungsten pins. Optimal orientation must be carried out even more accurately. This can be achieved by the use of alignment instruments like cross hair eyepieces. Caution is recommended when increasing the staining time to reach more high-contrast pictures, as surrounding areas or the whole cuticula might be discolored.

An additional feature allowing the upload of manually charted MAS patterns would further increase the usability of the database. Standardized patterns might considerably improve genus determination, as the influence of small structures would be reduced. Furthermore, MAS patterns in specimens with discolorations or other disturbances could be included. In particular this concerns pictures which were classified as C images due to disadvantageous features found in the larva, not because of image capturing problems. Such disturbances might interfere with the computerized analysis.

A number of more traditional morphological species indicators are located on the cuticula of larval Diptera, such as spinal bands and spiracles. While preparing the cuticula according to protocol, such indicators could automatically be included. If—due to close relatedness or other factors—the results of MAS analysis are not unambiguous, those other morphological characteristics might be used to substantiate species or genus identification. The MAS method thus complements traditional methods, rather than rivalling them.

MAS methods, of course, are not restricted to forensically important Diptera. Many of the over 200 known dipteran families might not be forensically important, but could be included in a MAS database for species determination in other fields of entomology. Furthermore, if the data base can be expanded, identification might not remain the only objective. Not all dipteran larvae live in the same habitat and have the same behavior. Muscle equipment of larvae might reflect such differences. An indication for further analysis of muscle equipment and behavior could be given by muscle attachment site pattern comparisons in different species. Obligatory parasitic larvae for instance might have a reduced number of muscles due to diminished motility. Confirmation of this statement, however, needs further studies of larvae representing different breeding behaviors. Such a general view could lead to new insight in ecological systems.

## 7. Conclusions

With the growth of the knowledge base and a complete description of all segments, there might not only arise more differences between species, but also some so far unknown similarities. In an ambitious project, a complete map could be drawn of all clusters in all accessible developmental stages of larvae. The tempting prospect would be that even a fragment of a larva would be sufficient to determine its species and developmental state. An increase in the precision of the software will therefore be essential, which in turn could lead to the ability to distinguish seemingly indistinguishable species (e.g., Piophilinae species).

Finally, with a broad knowledge base and the corresponding software, species determination in dipteran larvae would become a quick and performable task for every interested individual able to take a digital picture of the larval cuticula. No entomological training and/or piles of field guides and keys with endless details would be needed!

## Figures and Tables

**Table 1:** Pro and contra for automatised image processing application.

Aspects Favouring Automatized Image Processing	Challenges
- defined illumination conditions - simple patterns - easy acquisition of objects for knowledge base	- different object sizes - differences in alignment - differences due to biological variability

**Table 2:** Results of species determination. The results are listed alphabetically and given as a ratio of correctly identified larvae to the total number of larvae analyzed. The highlighted fields indicate trials in which not all larvae were identified correctly. Trials marked with a dash (/) could not be executed due to the lack of suitable images of the required quality.

No.	Species	Group A		Group B	
		**Trial**	**Result**	**Trial**	**Result**
01	*Calliphora vicina*	A1	14/15	B1	14/15
02	*Lucilia ampullacea*	/	/	B2	4/7
03	*Lucilia illustris*	/	/	B3	6/8
04	*Lucilia richardsi*	/	/	B4	7/7
05	*Lucilia silvarum*	/	/	B5	4/4
06	*Sarcophaga albiceps*	/	/	B6	10/10
07	*Sarcophaga argyrostoma*	/	/	B7	7/7
08	*Sarcophaga caerulescens*	/	/	B8	9/10
09	*Sarcophaga similis*	/	/	B9	5/6
10	*Wohlfarthia bella*	A10	3/3	B10	7/7
11	*Wohlfarthia indigens*	A11	2/2	B11	6/6
12	*Wohlfarthia nuba*	/	/	B12	3/3
13	*Wohlfarthia trina*	A13	2/2	B13	9/10
14	*Wohlfarthia villeneuvi*	/	/	B14	12/12

**Table 3:** Results of genus determination. The results are listed alphabetically and given as a ratio of correctly identified larvae to the total number of larvae analyzed. The highlighted fields indicate trials in which not all larvae were identified correctly. Trials marked with a dash (/) could not be executed due to the lack of suitable images of the required quality.

No.	Species	Group A		Group B	
		**Trial**	**Result**	**Trial**	**Result**
01	*Lucilia ampullacea*	/	/	B2	6/7
02	*Lucilia illustris*	/	/	B3	8/8
03	*Lucilia richardsi*	/	/	B4	3/7
04	*Lucilia silvarum*	/	/	B5	3/4
05	*Sarcophaga albiceps*	/	/	B6	6/10
06	*Sarcophaga argyrostoma*	/	/	B7	1/7
07	*Sarcophaga caerulescens*	/	/	B8	0/10
08	*Sarcophaga similis*	/	/	B9	3/6
09	*Wohlfarthia bella*	A10	3/3	B10	7/7
10	*Wohlfarthia indigens*	A11	2/2	B11	6/6
11	*Wohlfarthia nuba*	/	/	B12	3/3
12	*Wohlfarthia trina*	A13	2/2	B13	10/10
13	*Wohlfarthia villeneuvi*	/	/	B14	12/12

**Table 4:** Similarities between the references (extract). The results are listed alphabetically; the numbers given are correlation coefficients compared to the reference. Highlighted fields indicate trials in which the genus of adjacent references differed from the genus of the reference analyzed.

Reference	References of Adjacent Similarity		
Lucilia ampullacea	Lucilia richardsi	Lucilia silvarum	Lucilia illustris
	(0.7944)	(0.7843)	(0.7759)
Lucilia illustris	Lucilia richardsi	Lucilia silvarum	Lucilia ampullacea
	(0.7994)	(0.7882)	(0.7872)
Lucilia richardsi	Lucilia ampullacea	Lucilia illustris	Lucilia silvarum
	(0.8040)	(0.7930)	(0.7919)
Lucilia silvarum	Lucilia richardsi	Lucilia ampullacea	Lucilia illustris
	(0.7950)	(0.7890)	(0.7696)
Sarcophaga albiceps	Sarcophaga similis	Wohlfarthia indigens	Sarcophaga caerulescens
	(0.7137)	(0.7070)	(0.7061)
Sarcophaga argyrostoma	Sarcophaga caerulescens	Sarcophaga similis	Lucilia richardsi
	(0.7366)	(0.7305)	(0.7243)
Sarcophaga caerulescens	Sarcophaga similis	Wohlfarthia trina	Wohlfarthia villeneuvi
	(0.7612)	(0.7598)	(0.7489)
Sarcophaga similis	Sarcophaga caerulescens	Wohlfarthia indigens	Lucilia richardsi
	(0.7714)	(0.7526)	(0.7472)
Wohlfarthia bella	Wohlfarthia villeneuvi	Wohlfarthia trina	Wohlfarthia indigens
	(0.7671)	(0.7566)	(0.7382)
Wohlfarthia indigens	Wohlfarthia bella	Wohlfarthia villeneuvi	Wohlfarthia trina
	(0.7402)	(0.7343)	(0.7341)
Wohlfarthia nuba	Wohlfarthia indigens	Wohlfarthia trina	Wohlfarthia villeneuvi
	(0.7725)	(0.7492)	(0.7402)
Wohlfarthia trina	Wohlfarthia indigens	Wohlfarthia villeneuvi	Wohlfarthia nuba
	(0.7833)	(0.7758)	(0.7641)
Wohlfarthia villeneuvi	Wohlfarthia indigens	Wohlfarthia trina	Wohlfarthia bella
	(0.7976)	(0.7940)	(0.7792)

**Table 5:** Recognized problems and possible solutions.

Problem	Solution
The patterns contain many holes.	The combination of high-pass filter and threshold should be adjusted. The aim is to recognise each pattern point as closed, hollow form. Afterwards a dilatation algorithm is applied.
In holey patterns parts of the border may be missing. This affects the identification. Additionally, holey patterns may mislead interpretation of the layered image. The result is a reference pattern with poor properties.	
The patterns of the reference are too voluminous. This changes the correlation coefficient.	Guidelines for the generation of references need to be set. Firstly, no pattern with holes should be used. Furthermore, it is important to layer at least 10 patterns, to generate a valid reference. Additionally, a new tool should be developed to allow the rotation of patterns.
The pattern to be analysed is too voluminous.	A predefined area can be built around the geometric centre of the pattern point.
In this case the pattern often has holes. This changes the correlation coefficient.	This new pattern can be used instead of the pattern point.
Misinterpretation of central patterns.	Three possible solutions could be investigated: Firstly, it should be tested whether the central pattern can be ignored. If this is not possible it should be scaled in width. If this also does not yield any favourable results, it might be tested whether the central pattern can be split along the axis of symmetry.
The central pattern consists of two or more partial patterns. Those vary significantly in size and distance within one species.	
In addition, the patterns of species of the same genus show many similarities.	

## References

[1] Byrd J.H., Castner J.L. (2009). Forensic Entomology: The Utility of Arthropods in Legal Investigations.

[2] Greenberg B., Kunich J.C. (2002). Entomology and the Law. Flies as Forensic Indicators.

[3] Gennard D.E. (2007). Forensic Entomology: An Introduction.

[4] Lane R.P. (1975). Investigation into Blowfly (Diptera-Calliphoridae) Succession on Corpses. J. Nat. Hist..

[5] Catts E.P., Goff M.L. (1992). Forensic entomology in criminal investigations. Annu. Rev. Entomol..

[6] Carter D.O., Yellowlees D., Tibbett M. (2007). Cadaver decomposition in terrestrial ecosystems. Naturwissenschaften.

[7] Reiter C. (1984). Zum Wachstumsverhalten der Maden der blauen Schmeißfliege Calliphora vicina. Z. Rechtsmed..

[8] Anderson G.S. (2000). Minimum and maximum development rates of some forensically important Calliphoridae (Diptera). J. Forensic Sci..

[9] Kaneshrajah G., Turner B. (2004). Calliphora vicina larvae grow at different rates on different body tissues. Int. J. Leg. Med..

[10] Donovan S.E., Hall M.J.R., Turnerand B., Moncrieff C.B. (2006). Larval Growth Rates of the Blowfly, Calliphora vicina, Over a Range of Temperatures. Med. Vet. Entomol..

[11] Niederegger S., Pastuschek J., Mall G. (2010). Preliminary studies of the influence of fluctuating temperatures on the development of various forensically relevant flies. Forensic Sci. Int..

[12] Amendt J., Krettek R., Niess C., Zehnerand R., Bratzke H. (2000). Forensic entomology in Germany. Forensic Sci. Int..

[13] O’Flynn M.A., Moorhouse D.E. (1980). Identification of early immature stages of some common Queensland carrion flies. J. Aust. Entomol. Soc..

[14] Cantrell B.K. (1981). The Immature Stages of some Australian Sarcophaginae (Diptera: Sarcophagidae). J. Aust. Entomol. Soc..

[15] Reiter C., Wollenek G. (1983). Zur Artbestimmung der Maden forensisch bedeutsamer Schmeißfliegen. Z. Rechtsmed..

[16] Erzinçlioğlu Y.Z. (1985). Immature stages of British Calliphora and Cynomya, with a re-evaluation of the taxonomic characters of larval Calliphoridae (Diptera). J. Nat. Hist..

[17] Queiroz M.M.D., deMello R.P., Lima M.M. (1997). Morphological aspects of the larval instars of Chrysomya albiceps (Diptera, Calliphoridae) reared in the laboratory. Mem. Inst. Oswaldo Cruz.

[18] Wallman J.F. (2001). Third-Instar Larvae of Common Carrion-Breeding Blowflies of the Genus Calliphora (Diptera: Calliphoridae) in South Australia. Invertebr. Taxon..

[19] Sukontason K.L., Piangjai S., Boonsriwong W., Bunchu N., Ngern-klun R., Vogtsbergerand R.C., Sukontason K. (2006). Observations of the third instar larva and puparium of Chrysomya bezziana (Diptera: Calliphoridae). Parasitol. Res..

[20] Szpila K., Pape T., Rusinek A. (2008). Morphology of the first instar of *Calliphora vicina*, *Phormia regina* and *Lucilia illustris* (Diptera, Calliphoridae). Med. Vet. Entomol..

[21] Martin-Vega D., Gomez-Gomez A., Baz A., Diaz-Aranda L.M. (2011). New piophilid in town: the first Palaearctic record of Piophila megastigmata and its coexistence with Piophila casei in central Spain. Med. Vet. Entomol.

[22] Singh D., Garg R., Wadhawan B. (2012). Ultramorphological characteristics of immature stages of a forensically important fly *Parasarcophaga ruficornis* (Fabricius) (Diptera: Sarcophagidae). Parasitol. Res..

[23] Wells J.D., Sperling F.A.H. (2001). DNA-based identification of forensically important Chrysomyinae (Diptera: Calliphoridae). Forensic Sci. Int..

[24] Wells J.D., Pape T., Sperling F.A.H. (2001). DNA-Based Identification and Molecular Systematics of Forensically Important Sarcophagidae (Diptera). J. Forensic Sci..

[25] Boehme P., Amendt J., Disney R.H.L., Zehner R. (2010). Molecular identification of carrion-breeding scuttle flies (Diptera: Phoridae) using COI barcodes. Int. J. Leg. Med..

[26] Sukontason K.L., Piangjai S., Bunchu N., Chaiwong T., Sripakdee D., Boonsriwong W., Vogtsberger R.C., Sukontason K. (2006). Surface ultrastructure of the puparia of the blow fly, Lucilia cuprina (Diptera: Calliphoridae), and flesh fly, Liosarcophaga dux (Diptera: Sarcophagidae). Parasitol. Res..

[27] Anderson G.S. (2004). Determining time of death using blow fly eggs in the early postmortem interval. Int. J. Leg. Med..

[28] Crossley A.C. (1965). Transformations in Abdominal Muscles of Blue Blow-Fly Calliphora Erythrocephala (Meig) during Metamorphosis. J. Embryol. Exp. Morphol..

[29] Hooper J.E. (1986). Homeotic Gene-Function in the Muscles of Drosophila Larvae. EMBO J..

[30] Roberts M.J. (1971). Locomotion of Cyclorrhaphan Maggots (Diptera). J. Nat. Hist..

[31] Hanslik U., Schoofs A., Niederegger S., Heinzel H.G., Spiess R. (2010). The Thoracic Muscular System and Its Innervation in Third Instar Calliphora vicina Larvae. I. Muscles of the Pro- and Mesothorax and the Pharyngeal Complex. J. Morphol..

[32] Niederegger S., Spieß R. (2012). Cuticular muscle attachment sites as a tool for species determination in blowfly larvae. Parasitol. Res..

[33] Niederegger S., Miroschnikow A., Spieß R. (2013). Marked for life: muscle attachment site patterns in blowfly larvae are constant throughout development. Parasitol. Res..

[34] Niederegger S., Spieß R. (2014). Muscle attachment sites of *Phormia regina* (Meigen). Parasitol. Res..

[35] Martin-Vega D., Baz A., Diaz-Aranda L.M. (2012). The immature stages of the necrophagous fly, Prochyliza nigrimana: comparison with Piophila casei and medicolegal considerations (Diptera: Piophilidae). Parasitol. Res..

[36] Sukontason K., Sribanditmongkol P., Ngoen-klan R., Klong-klaew T., Moophayak K., Sukontason K.L. (2010). Differentiation between Lucilia cuprina and Hemipyrellia ligurriens (Diptera: Calliphoridae) larvae for use in forensic entomology applications. Parasitol. Res..

[37] Niederegger S., Szpila K., Mall G. (2015). Muscle attachment site (MAS) patterns for species determination in European species of Lucilia (Diptera: Calliphoridae). Parasitol. Res..

[38] Smith K.G.V. (1986). A Manual of Forensic Entomology.

[39] Szpila K., Amendt J., Goff M.L., Campobasso C.P., Grassberger M. (2010). Key for the identification of third instars of European blowflies (Diptera: Calliphoridae) of forensic importance. Current Concepts in Forensic Entomology.

[40] Szpila K., Hall M.J.R., Pape T., Grzywacz A. (2013). Morphology and identification of first instars of the European and Mediterranean blowflies of forensic importance. Part II. Luciliinae. Med. Vet. Entomol..

[41] Schumann H. (1971). Die Gattung Lucilia (Goldfliegen). Merkbl. Angew. Parasitenkd. Jena.

[42] Sonet G., Jordaens K., Braet Y., Desmyter S. (2012). Why is the molecular identification of the forensically important blowfly species Lucilia caesar and L. illustris (family Calliphoridae) so problematic?. Forensic Sci. Int..

[43] Szpila K., Richet R., Pape T. (2015). Third instar larvae of flesh flies (Diptera: Sarcophagidae) of forensic importance—Critical review of characters and key for European species. Parasitol. Res..

[44] Anton E., Niederegger S., Beutel R.G. (2011). Beetles and flies collected on pig carrion in an experimental setting in Thuringia and their forensic implications. Med. Vet. Entomol..

[45] Cherix D., Wyss C., Pape T. (2012). Occurrences of flesh flies (Diptera: Sarcophagidae) on human cadavers in Switzerland, and their importance as forensic indicators. Forensic Sci. Int..

[46] Grassberger M., Frank C. (2004). Initial Study of Arthropod Succession on Pig Carrion in a Central European Urban Habitat. J. Med. Entomol..

[47] Mᶏdra A., Frᶏtczak K., Grzywacz A., Matuszewski S. (2015). Long-term study of pig carrion entomofauna. Forensic Sci. Int..

[48] Matuszewski S., Szafalowicz M., Jarmusz M. (2013). Insects colonising carcasses in open and forest habitats of Central Europe: Search for indicators of corpse relocation. Forensic Sci. Int..

[49] Pohjoismäki J.L., Karhunen P.J., Goebeler S., Saukko P., Saaksjarvi I.E. (2010). Indoors forensic entomology: Colonization of human remains in closed environments by specific species of sarcosaprophagous flies. Forensic Sci. Int..

[50] Szpila K., Madra A., Jarmusz M., Matuszewski S. (2015). Flesh flies (Diptera: Sarcophagidae) colonising large carcasses in Central Europe. Parasitol. Res..

[51] Niederegger S., Szpila K., Mall G. (2016). Muscle attachment site (MAS) patterns for species determination in five species of Sarcophaga (Diptera: Sarcophagidae). Parasitol. Res..

[52] Martin-Vega D., Niederegger S. (2015). Larval muscle attachment site (MAS) patterns are a conserved character among Piophilini flies (Diptera, Piophilidae). Dtsch. Entomol. Z..

[53] Jähne B. (2002). Digitale Bildverarbeitung.

[54] Maschotta R. (2009). Merkmalslistenbasierte Kreuzkorrelationsmethoden für die medizinische Bildverarbeitung.

